# Evaluation of the Fatigue Behavior and Failure Mechanisms of 4340 Steel Coated with WIP-C1 (Ni/CrC) by Cold Spray

**DOI:** 10.3390/ma15228116

**Published:** 2022-11-16

**Authors:** Viorel Goanță, Corneliu Munteanu, Sinan Müftü, Bogdan Istrate, Patricia Schwartz, Samuel Boese, Gehn Ferguson, Ciprian-Ionuț Morăraș, Adrian Stefan

**Affiliations:** 1 Mechanical Engineering, Mechatronics and Robotics Department, Mechanical Engineering Faculty, “Gheorghe Asachi” Technical University of Iasi, 700050 Iasi, Romania; 2 Technical Sciences Academy of Romania, 030167 Bucharest, Romania; 3 Department of Mechanical and Industrial Engineering, Northeastern University, Boston, MA 02115, USA; 4 Kostas Research Institute, Northeastern University, Burlington, MA 01803, USA; 5 Army Research Laboratories, Aberdeen Proving Ground, Maryland, MD 21005, USA

**Keywords:** cold spray, coating, fatigue, SEM analysis, crack initiation, surface damage

## Abstract

Fatigue behavior of standardized 4340 steel samples uniformly coated with WIP-C1 (Ni/CrC) by cold spray was investigated. In particular, when a crack appeared at the interface between the base material and the coating, the cause of it as well as its shape and size were investigated. Fatigue loading was applied by alternating symmetrical cycles. Scanning electron microscopy was used to study the onset of failure and the subsequent propagation of cracks. The interface between the two materials performed well—in all samples, the initial crack propagation occurred on the surface of the base material, continuing into the coating material and in the interior of the base material. The fatigue durability curve of stress vs. number of cycles (S-N) presented a conventional form for a metallic alloy and the coating material had an influence only on the damage on the surface of the base material.

## 1. Introduction

In recent years, 4340 low-alloy steels have been used for their high mechanical strength in military, industrial and aerospace applications [1]. Specifically, 4340 contains Cr, Ni, V and Mo as alloying elements, which contribute to the improvement of fatigue resistance and hardness. Besides 4340 steels, other types of alloys such as AISI 4130, AISI 4140, AISI 4340, 300 M and D6AC also have high mechanical strength and are used in both the automotive and oil industries [2]. These materials are used in the components of machine parts subjected to high dynamic stresses such as torsion bars, sprockets, crankshafts and other parts that have high mechanical requirements [3]. Studies revealed that several carbide types in the alloy’s microstructure such as Metal2C, Metal7C3, Metal23C6 and Metal3C are formed [4,5]. A study also highlighted that the alloying elements affect the transformation of austenite by modifying the beginning and ending temperatures of the phase transformations [6]. In order to improve the properties of the materials, researchers have started assessing various types of coatings by different methods [7].

In order to protect against corrosion, researchers analyzed the coating surface at high temperatures. There are studies in the literature that focus more on characterizing the coating or the interface between the two materials (4340 steel and WIP-C1 (Ni/CrC) coating) than on the experimental part of analysis and highlighting the initiation and propagation of fatigue cracks [8].

Cold spray (CS) is a high-performance coating technique used for restoration and dimensional reconditioning of high-value assets with powders of metal alloys and metal–ceramic mixtures [9]. Other applications of the CS process consist of the use of pure elements [10], composite materials [11], some Al and Ti alloys [12], stainless steels [13] and super alloys of the Inconel 718 type [14]. During this deposition process, the metallic–ceramic powder particles are accelerated by a de Laval nozzle using preheated gases (He or N_2_) to high velocities in the range of 300–1200 m/s. The particles impact the surface and form a bonding layer on the surface of the base material. The coating process continues due to the particle–particle bonding of the powder in a layer-by-layer manner until the desired coating thickness is reached [15,16].

Chatha et al. [17] presented high deposition efficiency achieved in Cr_3_C_2_-Ni thermal spray coatings due to the high ductility of the Ni, using the following parameters: Oxygen flow rate 250 L/m, fuel (LPG), flow rate 60 L/m, air flow rate 600 L/m, spray distance 18 cm, fuel pressure 7.5 kg/cm^2^, oxygen pressure 9 kg/cm^2^ and air pressure 6 kg/cm^2^. Moreover, Ni and NiCr offer excellent oxidation and corrosion resistance at high temperatures and have similar coefficients of thermal expansion, thus minimizing the effect of stresses generated by varying thermal expansion.

CS coatings have been used to explore improvements in fatigue life and strength of substrate materials. In most cases, coating systems consist of dissimilar materials [18,19,20,21,22,23,24,25,26,27,28,29,30,31,32,33,34,35,36], but similar systems have also been explored [37]. In some systems, fatigue cracks initiate primarily from the defects within the substrate, and only secondary cracks develop in the coating. This is particularly true if the coating is primarily in a compressive state [24,25]. In other systems, cracks initiate in the interface of the coating and the substrate. Depending on the condition of the residual stresses and the strength of bonding, cracks can propagate into the coating, or the substrate. In successful applications, the crack propagation is delayed by the systematic improvements that the CS coating provides.

Fatigue behavior of cold sprayed coatings is influenced by the strength of the coating–substrate interface, residual stress in the coating and the substrate, material properties of the coating and substrate and surface conditions including the roughness of the substrate [18]. In some applications, particle impacts lead to excessive roughening of the substrate surface which act as crack-initiation sites leading to reduction in fatigue characteristics of the base material [19,21]. In other applications, adhesion strength in the coating–substrate interface and compressive stresses developed in the coating and potentially in the substrate lead to improvements [20].

Material properties and manufacturing methods of the powders can also affect the fatigue strength of the coating systems. These differences can often be traced to the bonding quality of the coating to the substrate, and to the porosity in the coating induced due to different material systems and particle properties such as shape or strength [22,23]. Addition of hard particles to metal powders can improve the coating quality by reducing the porosity in the coating, increasing the bond strength between the coating and the substrate, work hardening the substrate due to shot peening. In some cases, hard particles that are embedded in the coating can arrest crack growth or propagation [26,27].

Initial preparation of the substrate surface also has an influence on the bond strength between the coating and the substrate [28,29,30,31,32] and thus it can affect the fatigue characteristics. Surface preparation techniques include grit blasting, polishing and grinding [28,29,30,31,32,33], shot peeing with hard particles [34] and blasting the surface with the same particles as the coating at low impact angles [38]. Shot peening of the specimens prior to CS coating has been shown to enhance the fatigue strength [34].

Other studies related to fatigue of CS-coated materials are presented by Su et al. [39], who compare the fatigue and wear performance of samples with Ti- and Cr-based coatings, and their specific cracking mechanisms. Gu et al. [40] present durability diagrams showing the effect of hard TiN film on the fatigue strength of AISI D2 tool steel. Puchi-Cabrera et al. [41] evaluated the fatigue behavior of 316 L stainless steel coated with a hydrogenated, amorphous carbon. In that case, the fractographic analysis after the fatigue stress showed that the cracks were nucleated on the surface of the coating material and propagated into the substrate.

Ozdemir et al. [42] performed high-speed depositions of tantalum powders using CS on a 4140 steel substrate. The study demonstrated that CS is a cost-saving method for repairing different types of machine parts. The effect of coatings on fatigue behavior was also investigated by Rhys-Jones et al. [43], concluding that the application of the coating layer leads to loss of strength compared to uncoated materials.

Dongming et al. [44] presented the fatigue behavior of several coated titanium alloys. For some alloys, such as Ti-6Al-4V, a decrease in fatigue strength was found in the coated samples. On the other hand, Ti-Al-based alloys present a slight improvement in fatigue strength for coated samples. In the case of 4340 low-alloy steel, combined-loading fatigue reliability research highlighted the behavior at combined stress and the way in which the crack is initiated and propagated [45,46]. As a result, in order to improve other properties such as wear behavior and high temperature resistance, for each specific coated alloy, both the behavior at variable stresses (where applicable) and the possible change in the base material properties must be studied.

The aim of this work is to study the fatigue behavior of the base material–coating material assembly including morphological analysis of crack initiation at the interface between the two materials.

## 2. Materials and Methods

### 2.1. Coating Process

The base material used for the fatigue test was AISI 4340 alloy steel which is a low-alloy steel containing mainly 0.4% C, 0.8% Cr, 0.25% Mo and 1.8% Ni. The material was delivered with a mild hardening having a tensile strength, σ_UTS_, of 890 MPa and a yield strength of 785 MPa. After cleaning, the specimens were coated with a WIP-C1 powder by using a VRC Gen-III cold spray machine (VRC Metal Systems, LLC., Box Elder, SD, USA). The parameters used for spraying the powder mixture are given in Table 1. The powder mixture is commercially known as WIP-C1 [17].

Before the surface was coated with WIP-C1 powder, a thin layer of bond coat that consists of WIP-BC1 powder was applied on the surface with 60° nozzle orientation. This operation has multiple effects—shot-peen, clean and roughen the surface, in addition to creating a thin layer of WIP-BC1 coating. The cylindrical specimens were mounted on a lathe and the WIP-C1 coating was applied by moving the nozzle axially, while holding it normal to the specimen axis. The spray conditions used to deposit the coatings are given in Table 1 and are similar to those in previous work [47].

### 2.2. Samples and Equipment Used for Tensile Testing

**Figure 1:**
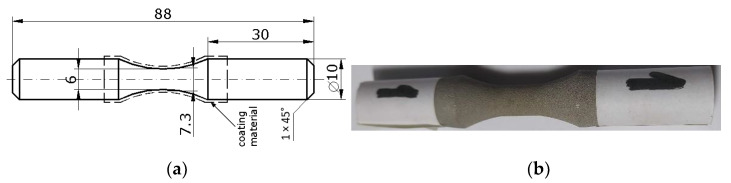
Details of tensile test sample: (**a**) Sample shape and dimensions (mm), (**b**) coated sample.

Before samples are tested for fatigue, certain characteristics of the material from which they are made must be determined. The static tensile characteristics of the material including its yield strength which affects its fatigue characteristics are determined according to the ASTM E8/E8M-16 standard [48]. The Instron 8801 universal machine (Wycombe, England) was used for the static and fatigue tests, which also has the possibility of dynamic fatigue tests. The frequency used for fatigue was 20 Hz, the maximum possible frequency of the testing machine being 25 Hz. The tested tensile samples had the dimensions and shape shown in Figure 1a,b.

### 2.3. Methodogy of Fatigue Analysis Performed on Coated Samples

For fatigue analysis, ten of the coated 4340 samples were tested, whereas sample A was used for a static tensile test. The tests were carried out according to the ASTM E466-15 standard [49], the stress being applied after a symmetrical alternating cycle. As a result, the cycle asymmetry coefficient was R = −1. Fatigue behavior analysis must be carried out in such a way that the values of the initial maximum stress remain in the elastic range, lower than the yield limit of 852 MPa. Starting with high stress values, the first value of the maximum normal stress was calculated as follows: σ_max_ = 0.748 × σp_0_._2_ = 637 MPa.

The value of 5 million planned cycles was chosen as a reference. If, for a certain stress, after 5 million cycles the sample did not break, the test machine was stopped. The H and G samples did not break even after 5 million cycles. The other samples broke at the stress levels and numbers of cycles presented in Table 2.

The SEM images highlighting the fracture surfaces of the samples tested for fatigue (discussed in the Results section) show aspects of the coating material in different areas. Due to the fact that ε_base_ > ε_coating_, the materials exhibit different levels of deformation. Since the two materials deform differently, a significant deterioration of the coating material was observed and large deformations occurred at higher values of the stress cycles.

## 3. Results

### 3.1. SEM Analysis of 4340 Steel Base Material

**Figure 2:**
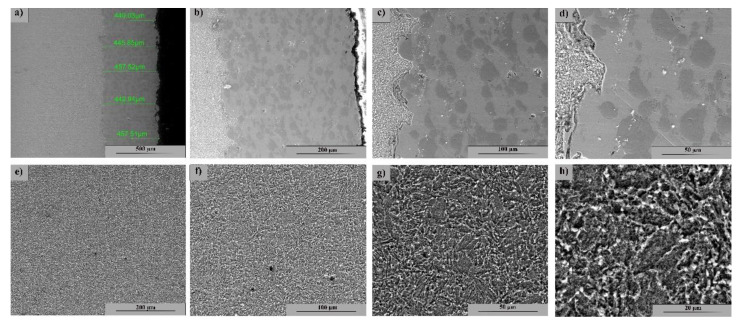
SEM image of the cross-section at the interface between the base and the coated materials: (**a**–**d**): Cold spray Ni/CrC layer—(**a**) 200×; (**b**) 500×; (**c**) 1000×; (**d**) 2000×; (**e**–**h**): 4340 microstructure base material—(**e**) 500×; (**f**) 1000×; (**g**) 2000×; (**h**) 5000×.

Figure 2 highlights the SEM image of the coated sample section as-coated (not tested). The coating is uniform, with no porosity; it is adherent to the substrate and has an approximate thickness of 450 μm.

Figure 2a–d show the layer deposited by the CS method at various magnifications, where a structural uniformity with coarse grain size and two distinct phases corresponding to the powders used is observed. The base material (Figure 2e–h) shows a uniform ferritic–pearlitic structure with a fine grain size without defects and isolated inclusions.

### 3.2. Fracture Analysis at Static Loading

**Figure 3:**
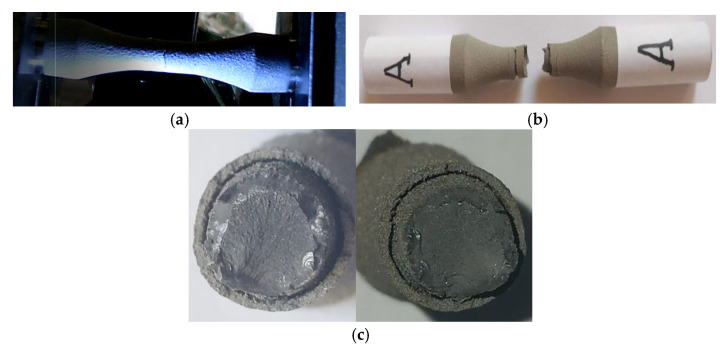
Sample A—static tensile test. (**a**) View of the sample during the tensile test. (**b**) Sample broken in static tensile test. (**c**) Front views of the fracture surfaces of the tensile specimen.

**Figure 4:**
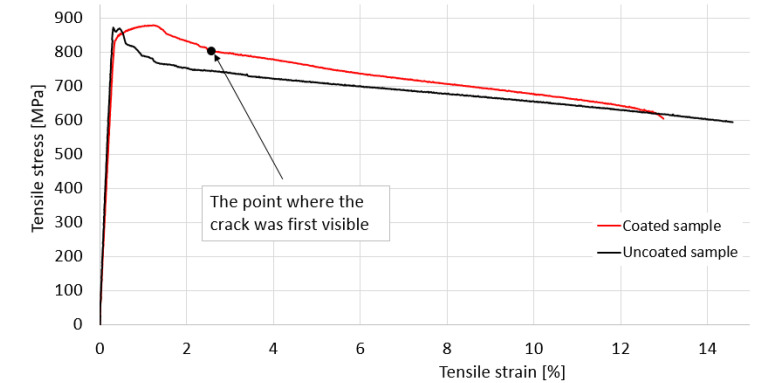
Characteristic stress–strain curve, specific to the coated sample and uncoated samples.

Images from the static tensile test are shown in Figure 3, and include the specimen immediately after the macrocrack becomes visible (Figure 3a), the broken specimen (Figure 3b) and the fracture surfaces (Figure 3c). Figure 4 shows the point determined by the visible macrocrack (Figure 3a) on the stress–strain curve. Both from the examination of the fracture surfaces of the static tensile analysis and from the study of the stress–strain variation given by the characteristic curve, the following conclusions are highlighted: (i) The way in which stress varies in relation to the strain, presented in Figure 4, is mainly due to the behavior of the base material. The coating material does not greatly influence the shape of the stress–strain curve; (ii) the elastic region reaches the maximum stress, thus reducing deformations in the elastic field; (iii) after recording the ultimate tensile strength (σ_uts_) the stress–strain variation decreases, registering a significant elongation of the sample, with no apparant hardening zone; (iv) with a total elongation of 13% at breakage, the base material presents significant plastic deformations, which is also visible in Figure 3b; (v) the coating material has significant deterioration due to the brittle behavior at high elongations; (vi) the fracture surfaces for both the coating material and the base material are approximately perpendicular to the geometric axis of the sample (Figure 3b,c) and are perpendicular to the direction of loading. Consequently, both materials exhibit brittle behavior under tensile stress; (viii) the coating material detached from the base material at the time of breaking the test samples; in the area of breakage there is a separation between the coating and the base material, with no contact between them; (ix) the yield limit (σp_0_._2_) was determined to be 852 MPa, a value taken into account when calculating the first maximum fatigue stress value.

**Figure 5:**
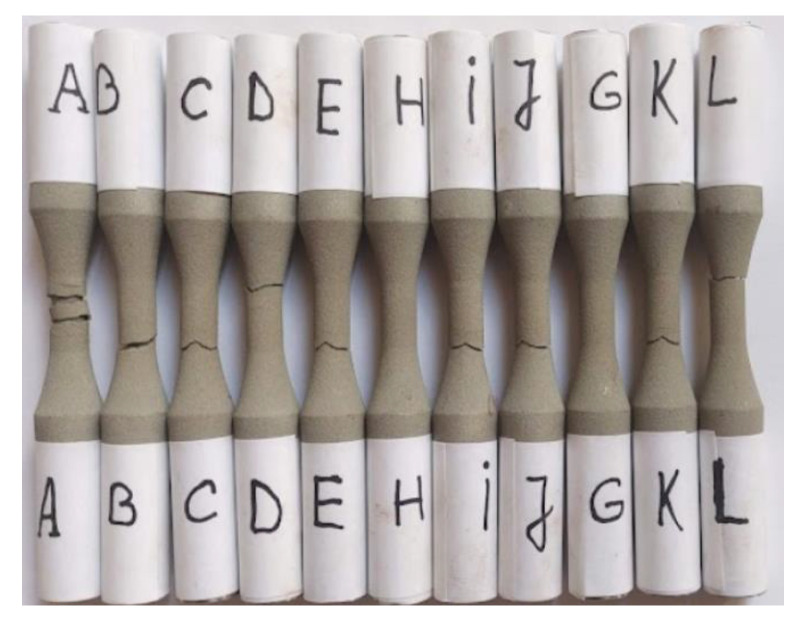
Samples after fatigue testing: A: static tensile sample; B–L: fatigue tested samples.

The present work covered analysis of ten samples (Figure 5—B to L), tested for axial-symmetrical tensile fatigue. Only eight of the ten samples were broken—samples H and G did not break even after 5 million cycles. Observations about each sample are presented in detail below. The samples with close values of the maximum loading stress are specified and discussed together.

### 3.3. Samples B and C (σ_max_ (MPa); N), (637; 3794) and (530; 15,726)

#### 3.3.1. Macroscopic Observations

**Figure 6:**
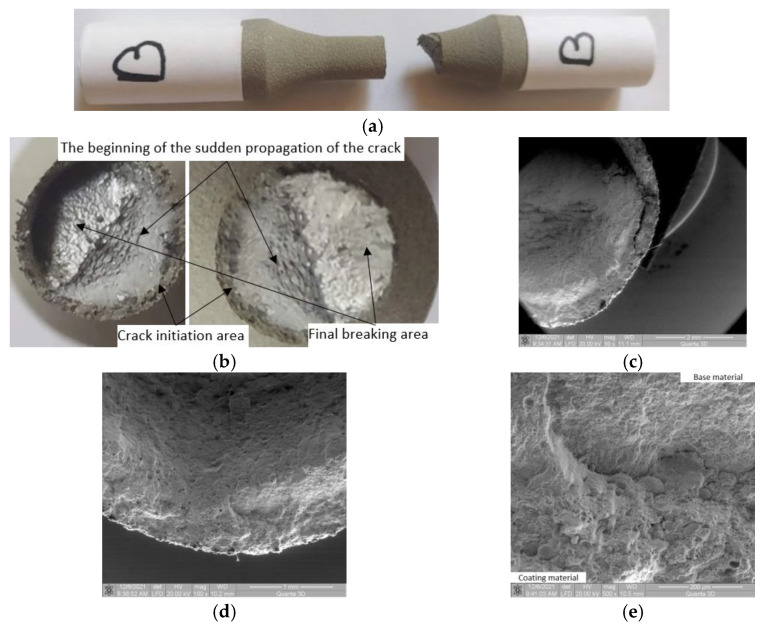
Macroscopic and microscopic images of broken surfaces for samples B and C. (**a**) Specimen B: Side view of the broken sample. (**b**) Specimen B: Macroscopic images of surfaces resulting from breakage. (**c**) Specimen B: SEM image (50×) of the broken surface at plane deflection. (**d**) Specimen B: SEM image (100×) of the interface area. (**e**) Specimen B: SEM image (500×) with damage in the interface area.

As was observed for all 4340 coated samples in the fatigue tests, towards the end of the crack propagation area, the cracked surface moves from the perpendicular plane to the test direction and goes by an angle of approximately 45° (Figure 6a–c). It was discovered that the fracture front exhibits resistance throughout its line of progression. In the unbroken area, the accumulated stresses are very high due to the fact that the section area is much smaller than the initial one, and the propagated crack constitutes a powerful stress concentrator. As a result, the plastic deformations in the unbroken area are high and almost the entire region is plastically deformed. In these conditions of stress asymmetry and large plastic deformations, the crack front is oriented in the direction of the minimum resistance encountered.

#### 3.3.2. Microscopic Observations

Significant damage occurs in the coating material in the moment the crack front propagation changes direction (Figure 6c). The crack initiation area is at the surface of the base material, at the interface between the base and coating material. At the interface, the formation of a large crack is not observed (Figure 6d,e). There is significant detachment and damage of the coating material in the initial area of crack deflection (Figure 6c). In the area of crack initiation (at the interface—Figure 6d,e), significant damage is found in the coating material. Greater damage is at the interface between the coating material and the base material—a confirmation that the crack begins at the surface of the base material and extends into the coating material. This is possible due to high stresses, in which case there was no crack-type detachment between the two materials. This characteristic implies a serious coating material deterioration.

### 3.4. Samples D and E (σ_max_ (MPa); N), (496; 26,688) and (424; 59,291)

#### 3.4.1. Macroscopic Observations

**Figure 7:**
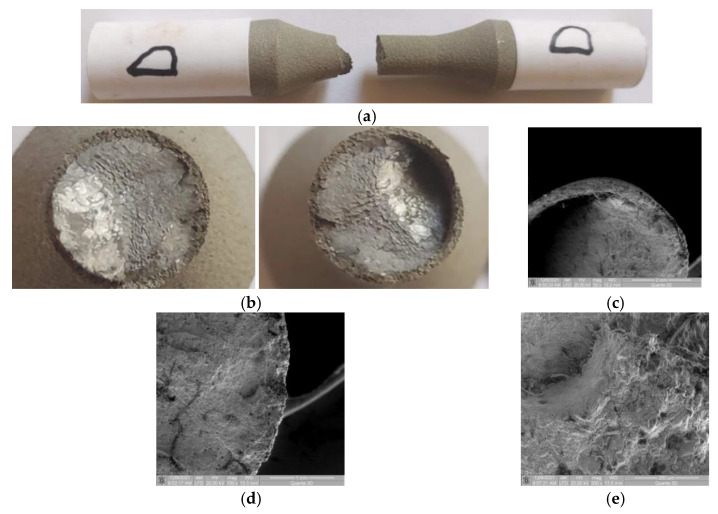
Macroscopic and microscopic images of fracture surfaces for samples D and E. (**a**) Specimen D: Side view of the broken sample. (**b**) Specimen D: Macroscopic images of surfaces re-sulting from breakage. (**c**) Specimen D: SEM image (50×) of the broken surface at plane deflection. (**d**) Specimen D: SEM image (100×) of the interface area. (**e**) Specimen D: SEM image (500×) with damage in the interface area.

For these samples, the angular deflection of the fracture surface is observed in Figure 7a–c. The stable fatigue propagation area of the crack is bigger (Figure 7b) because the stress value is lower than in the previous samples. From a macroscopic point of view, the coating material damage is observed only in the final fracture area. No significant damage was found at the moment the crack propagation changed direction (Figure 7c).

#### 3.4.2. Microscopic Observations

Due to the lower stresses, the damage to the area of the beginning of cracked surface deflection (Figure 7b,c) is smaller than in the previous samples. In the area of crack initiation, the same detachment from the coated material is observed (Figure 7d,e). Moreover, in the coated material, an intergranular radial crack is observed, which starts from the fracture initiation area (Figure 7d). Figure 7e presents average detachment from the coated material (as seen in the previous samples). The inter/intragranular cracks from the initiation area are much smaller than those of samples B and C.

### 3.5. Samples I and J (σ_max_ (MPa); N), (389; 91,986) and (371; 119,450)

#### 3.5.1. Macroscopic Observations

**Figure 8:**
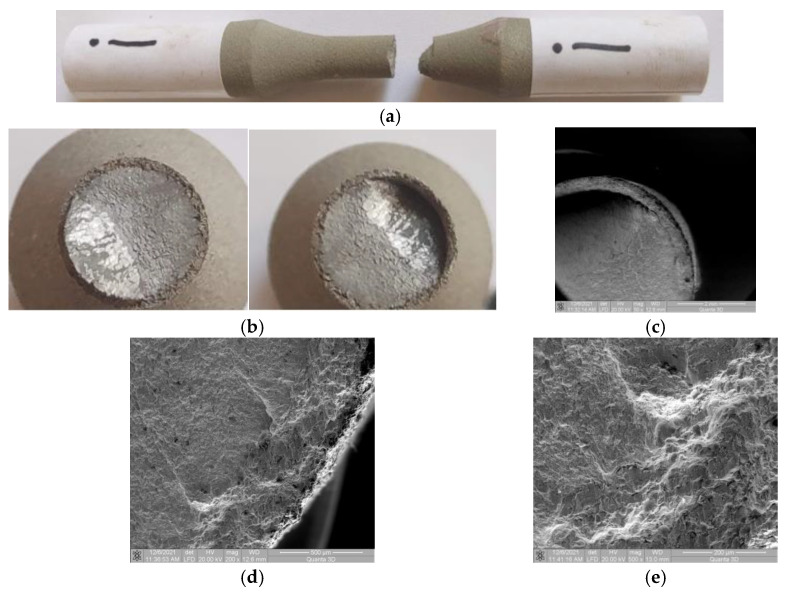
Macroscopic and microscopic images of broken surfaces for samples I and J. (**a**) Specimen I: Side view of the broken sample. (**b**) Specimen I: Macroscopic images of surfaces resulting from breakage. (**c**) Specimen I: SEM image (50×) of the broken surface at plane deflection. (**d**) Specimen I: SEM image (200×) of the interface area. (**e**) Specimen I: SEM image (500×) with damage in the interface area.

As the stress value decreases, the stable propagation area of the fatigue crack increases, and the area where the angled deflection of the cracked surface occurs becomes smaller (Figure 8a,b). The destruction of the cylinder formed by the coating material occurs only in the final area of the damage (Figure 8b,c). There are distinct regions of coated material and base material in the beginning of the crack front deflection area, but in the cracked surface deflection area, a detachment of the coating material from the base material is observed (Figure 8c). In this area, the large plastic deformation of the base material cannot be matched by the coating material.

#### 3.5.2. Microscopic Observations

In the area of fatigue crack initiation (at the surface of the base material—Figure 8d,e), no significant damage to the coating material is found. The propagated crack front through the coating material is not straight, having certain deviations from the approximately flat surface. It was found that the deviation of the crack front from perpendicularity to the applied force starts at the interface between the base material and the coating material. Apart from the crack initiation area, no inter- and/or intragranular cracks were observed in the rest of the coated material.

### 3.6. Samples K and L (σ_max_ (MPa); N), (361; 190,536) and (353; 291,111)

#### 3.6.1. Macroscopic Observations

**Figure 9:**
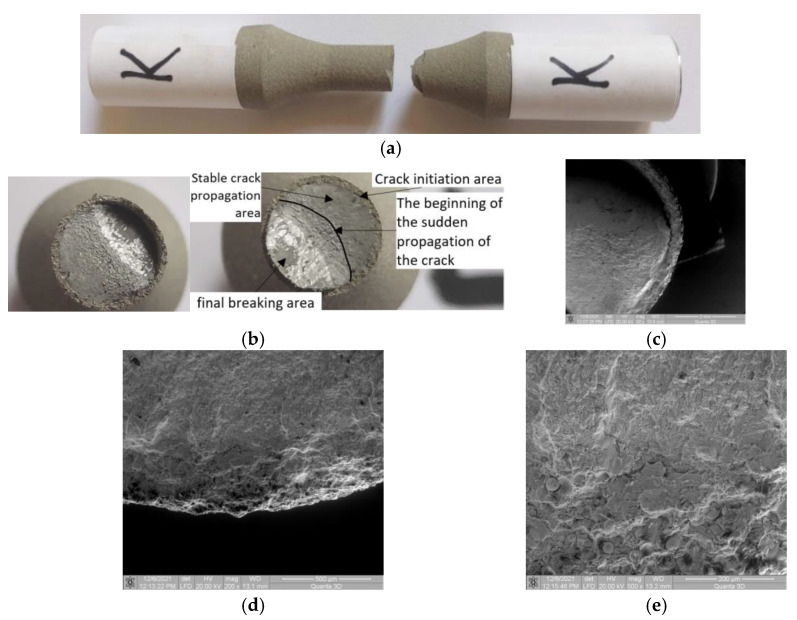
Macroscopic images of broken surfaces for the samples K and L. (**a**) Specimen K: Side view of the broken sample. (**b**) Specimen K: Macroscopic images of surfaces resulting from breakage. (**c**) Specimen K: SEM image (50×) of the broken surface at plane deflection. (**d**) Specimen K: SEM image (200×) of the interface area. (**e**) Specimen K: SEM image (500×) with damage in the interface area.

In the case of 4340 coated materials, all analyzed samples showed changes in the crack front direction at an angle of approximately 45°, relative to the initial crack front, which was perpendicular to the stress direction. This is due to the characteristics of the base material which, at a certain stress value, shows significant plastic deformation. Thus, a more intense displacement of dislocations is achieved, which can form a barrier to the propagation of fatigue cracks. In the case of the samples K and L, a change in the direction of crack propagation front is seen. The area of deflection is smaller and there is a detachment between the coating material and the base material at the beginning of the deflection area of the fracture surfaces. The area of stable propagation of the crack is clearly highlighted; the flat and perpendicular surface to the direction of stress also presents a surface of unstable crack propagation, which is near to the deflection of the crack front area (Figure 9c). In the crack deflection area, the same detachment is observed between the coated and the base material.

#### 3.6.2. Microscopic Observations

Figure 8d shows that in the area of crack initiation there is no damaged surface between the base and coated material. However, Figure 8e shows a small detachment of the coated material from the surface of the base material. This detachment is minor and it is influenced by the microstructure of the coated material in correlation to the initialization of the crack front. For the last samples, due to the low stress values, the fracture of the coated material was induced by the large crack opening in the base material. In this area, the coated material behaves well, with no inter/intragranular cracks or detachment from the material.

### 3.7. Samples G and H (σ_max_ (MPa); N), (318; 5,000,208) and (346; 5,000,099)

**Figure 10:**
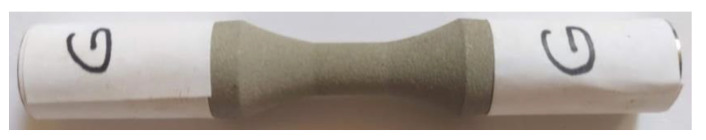
Sample G—unbroken after over 5 million cycles. σ = 318 MPa, *n* = 5,000,208 cycles.

**Figure 11:**
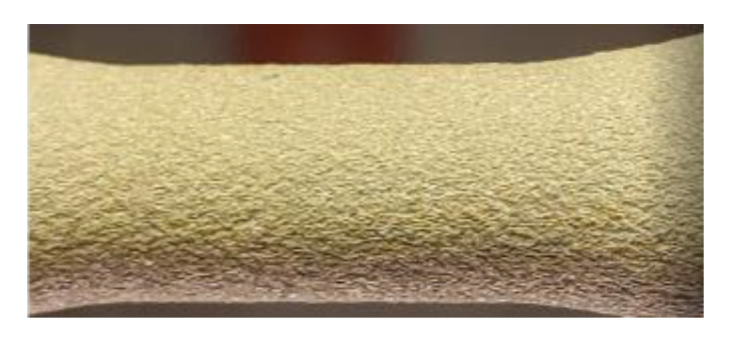
Macroscopic view of the surface of sample G (4340) after 5 million cycles.

**Figure 12:**
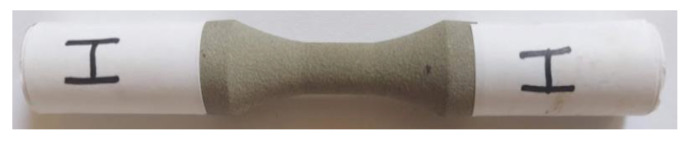
Sample H—unbroken after over 5 million cycles; σ = 346 MPa, *n* = 5,000,099 cycles.

**Figure 13:**
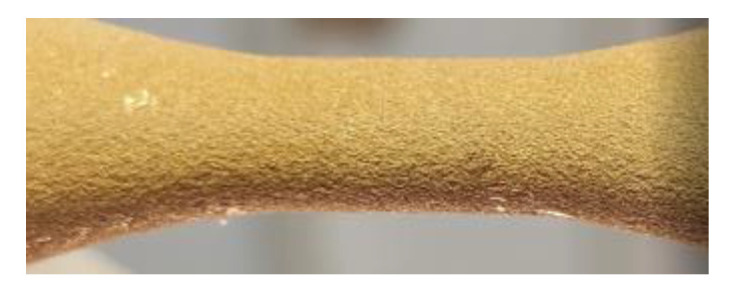
Macroscopic view of the surface of sample H (4340) after 5 million cycles.

Samples G and H were loaded at much lower stresses relative to the yield strength. The stress value was so low that the samples did not break even after 5 million cycles. The external surface of the coated material was studied from the macroscopic point of view (Figure 10, Figure 11, Figure 12 and Figure 13). No damage, cracks or detachment of the coated material were found at the surface.

Note: At a load of 346 MPa, sample H did not break even after 5,000,099 cycles while sample L, tested to 353 MPa, was broken after only 291,111 cycles. As a result, it was not possible to obtain a higher number of cycles at breakage, because the test should have been between approximately 353 MPa and 346 MPa, being a very small area in terms of test load. Considering the distribution of fatigue fracture and the semi-fragile comportment of the analyzed material assembly, it is concluded that this is the real behavior of the 4340 coated with Ni/CrC. If the test load is reduced below a certain stress (346 MPa), the material withstands high values in terms of the number of stress cycles, and if the fatigue working load exceeds a certain value (approximately 350 MPa), the material breaks off after a number of cycles—estimated around hundreds of thousands.

### 3.8. Microstructure of Samples K and H after Fatigue Test

**Figure 14:**
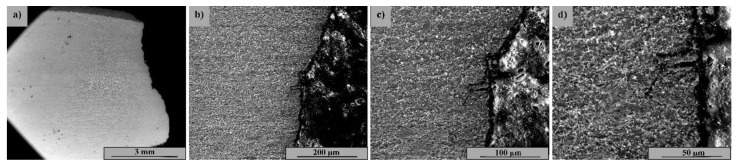
Microstructures from sample K in the breakage area: (**a**) Macroscopic view; (**b**) 500×; (**c**) 1000×; (**d**) 2000×.

**Figure 15:**
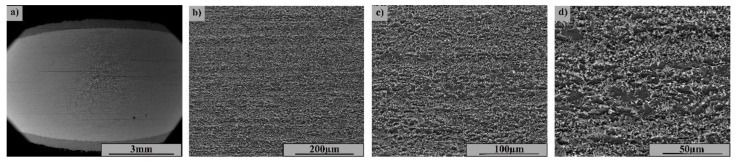
Microstructures in sample H fatigue stressed and unbroken: (**a**) Macroscopic view; (**b**) 500×; (**c**) 1000×; (**d**) 2000×.

The microstructure and cross-sectional breakage appearance of the K and H samples are shown by electron microscopy in Figure 14 and Figure 15. Figure 14a–d show crack propagation in the substrate and not at the interface between the coating and the base material, with the crack propagation occurring in a predominantly pearlitic zone with increased brittleness. The structure of the base material (Figure 1) shows a fibrous structure specific to the lamination process, with grains in ferrite and perlite strings.

In the region of the crack zone (Figure 14d), fine equiaxial grains are evident due to recrystallization processes during fatigue testing. The breakage is intracrystalline and preceded by microcracks at the boundary between the grains. In the case of sample H (Figure 15a–d), which after more than 5 million cycles has not cracked, the microstructure shows a fibrous appearance with ferrite and perlite grains in the direction of stress, without cracks or other local defects.

### 3.9. Wӧhler Diagram for Fatigue Tested 4340 Coated Samples 

**Figure 16:**
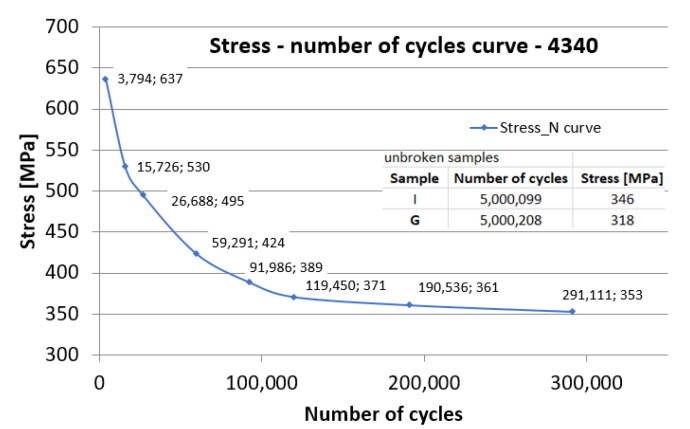
Wӧhler diagram for fatigue tests of the 4340 steel coated with WIP-C1 powder.

The Wӧhler diagram (stress vs. number of cycles—Figure 16) is based on the stress values at the number of cycles until fracture in cyclic fatigue tests. Samples H (346 MPa) and G (318 MPa) did not break after 5 million cycles. The layout of the diagram is a conventional one for a metallic material, with semi-brittle behavior at break, having an estimated fatigue limit around 346 MPa.

## 4. Summary and Discussion

In this work, the mechanism of material failure due to fatigue loading of a coating system formed by the 4340 steel and Ni/CrC coating deposited by CS is described. Samples of AISI 4340 alloy steel coated with WIP-C1 (Ni/CrC) powder by CS were tested for their fatigue resistance. In addition, the stress range in which the coated parts can be used in cyclical loading without damage to the base and coating materials is identified.

The deposition shows no geometric deviation of the deposited layer, and in addition it adheres well to the base material (Figure 1). This figure also shows that the surface area of the base material (AISI 4340 alloy steel) was partially affected by the intrusion of the coating material into base material surface.

In systems using ceramic coatings, the fatigue behavior of the interface between the coating material and the substrate material is important. Various researchers [24,25,37] have explored this behavior. Depending on the materials chosen (substrate and coating), different findings of how and where crack initiation and propagation occur have been obtained. In many of the studies, it is stated that cracks were primed at the interface between the two materials, meaning that a detachment of the coating material from the base material takes place, after which the crack propagates, most often, first into the coating material. In our case, it was found that, as a result of the coating process, the metal surface of the substrate was damaged. As a result, for all the samples, the initial crack appeared on the surface of the base material and extended into the sample and the coating material.

There is a significant jump in the number of cycles from a broken sample to an unbroken sample within a range of only 7 MPa. It is interesting to note that while fatigue loading can be attributed to the cracks that initiate from the interface into the base material, a threshold load exists below which this effect is not observed.

## 5. Conclusions

The following conclusions can be drawn from the fatigue test and the microscopic and macroscopic observations:

- For high stress loading, close to the yield strength value, significant damage of the coating layer is found at the interface with the base material. These failures consisted of detachment of coating material as well as microcracks, which appeared near the area of crack initiation.
- At high stress values, the deflection of the cracked surfaces at certain angles is observed. In the initiation region of this deflection, where the deformation (especially the plastic ones) is large, the coating material detaches from the base material. Additionally, the area of stable crack propagation in the base material has an uneven appearance.
- Given that, at high stresses, the damage to the coating material is significant, it is not recommended to use coated and fatigue-strained components above 370 MPa. If the stress values are between 350 MPa and 370 MPa, the damage to the coating material is reduced, and, for medium durability, the components can be used within this range of stresses without affecting the base material and coating assembly.
- At fatigue stress levels below 340 MPa, no damage occurred in the tested base and the coating over 5 million cycles. Therefore, it is concluded that parts made of AISI 4340 alloy steel and coated with WIP-C1 (Ni/CrC) powder using CS can be expected to operate with high durability if the applied fatigue stresses do not exceed 340 MPa, even considering the presence of the stress concentrators.

## Figures and Tables

**Table 1:** Cold spray (CS) deposition parameters used to prepare the fatigue specimens.

Parameter	Value
Gas	Nitrogen
Pressure	6.2 MPa (900 psi)
Temperature	675 °C
Nozzle ID	WC NZL0060
Nozzle throat size	2 mm
Powder feeder speed	10 rpm
Powder feeder gas flow	105 slm
Standoff distance	25 mm
Spray angle	90 deg.
Nozzle traverse speed	250 mm/s
Nozzle step distance	0.25 mm
Layer thickness	0.127 mm
Target coating thickness	0.508 mm
Powder	WIP-C1
Bond coat	WIP-BC1 and 60°

**Table 2:** Maximum stress (σ_max_) and the number of cycles up to failure (N), obtained for Ni/CrC coated 4340 steel samples.

Sample No.	σ_max_ (MPa)	N
B	637	3794
C	530	15,726
D	495	26,688
E	424	59,291
I	389	91,986
J	371	119,450
K	361	190,536
L	352	291,111

## Data Availability

Not applicable.
